# Rare Variation of Accessory Maxillary Ostium

**DOI:** 10.7759/cureus.11921

**Published:** 2020-12-05

**Authors:** Alhanouf AlQabbani, Razan Aldhahri, Abdulrahman Alhumaizi

**Affiliations:** 1 Otolaryngology - Head and Neck Surgery, King Abdulla Bin Abdulaziz University Hospital, Riyadh, SAU; 2 Otolaryngology - Head and Neck Surgery, King Faisal Specialist Hospital and Research Centre, Riyadh, SAU

**Keywords:** accessory maxillary ostium, rhinosinusitis, paranasal sinus, principle maxillary sinus ostium, nasal fontanelle

## Abstract

Accessory maxillary ostium (AMO) is one of the anatomical variations in the maxillary sinus. It can be present in different sizes, shapes, and locations. We here reported a case of a rare variation of AMO with large size in a patient with chronic rhinosinusitis. It is important to identify the presence of AMO especially in patients with chronic rhinosinusitis as it can lead to mucus recirculation and disease persistence.

## Introduction

Accessory maxillary ostium (AMO) is one of the anatomical variations in the maxillary sinus. It is usually located in the fontanelle which is the membranous part of the lateral nasal wall located in the middle meatus; between the uncinate process and inferior meatus. The fontanelle is divided by the uncinate process into anterior nasal fontanelle (ANF) and posterior nasal fontanelle (PNF) [1]. AMO has been reported to be located in ANF, PNF, or rarely in hiatus semilunaris [2,3]. AMO is presented as round or oval in shape, parallel to the vertical plane of the lateral nasal wall. In contrast to principle maxillary ostium which is hiding behind the uncinate process, AMO can be seen easily during nasal endoscopic examination [2]. It is reported to be present in 30% of the patients diagnosed with chronic maxillary sinusitis and in 10-20% of normal individuals [4,5].

For the purpose of adding more information about the AMO, we present this case report about a rare variation of the AMO.

## Case presentation

**Figure 1:**
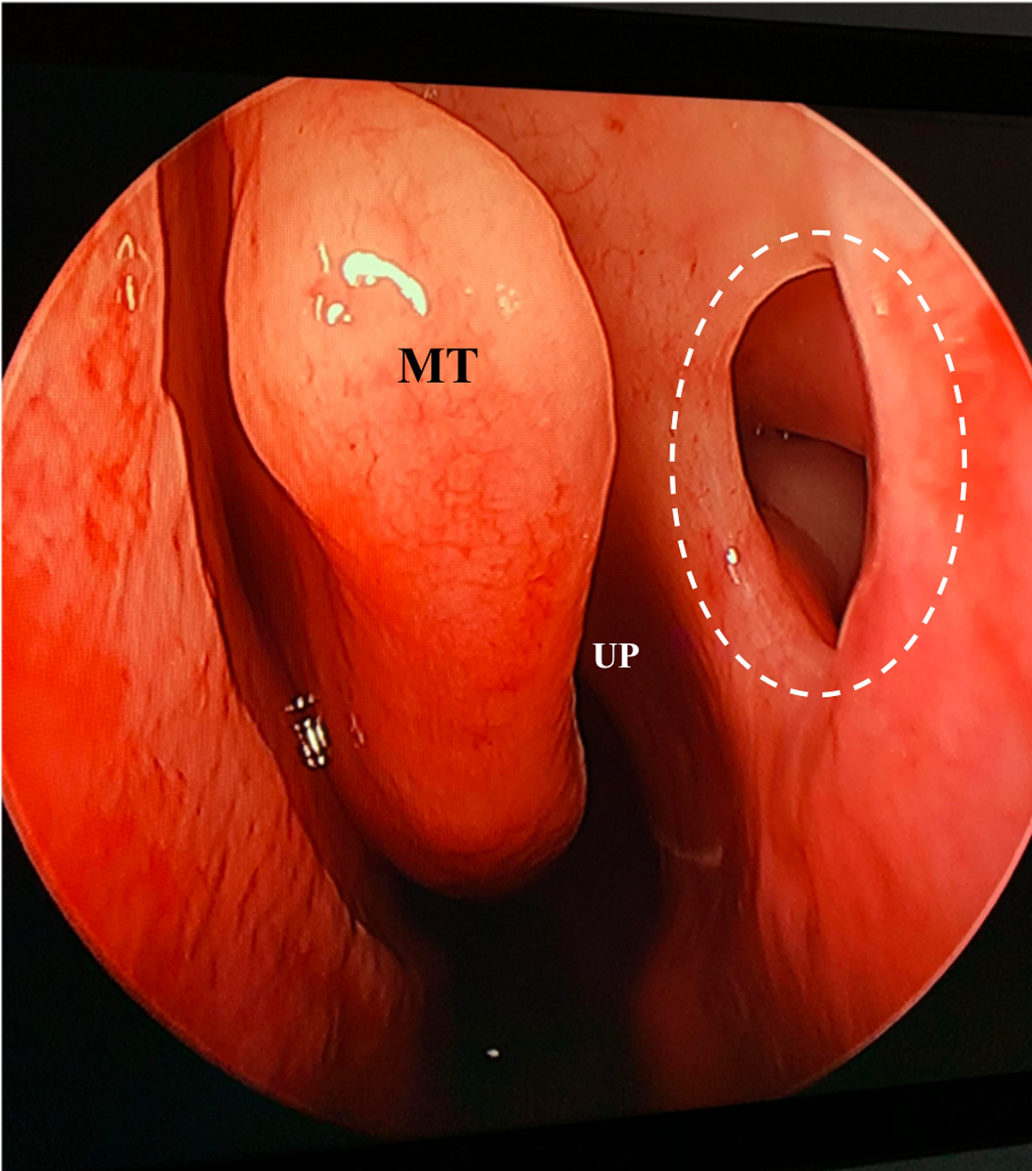
Left nasal endoscopy shows left AMO located in the anterior fontanelle. MT: middle turbinate, UP: uncinate process, AMO: accessory maxillary ostium.

**Figure 2:**
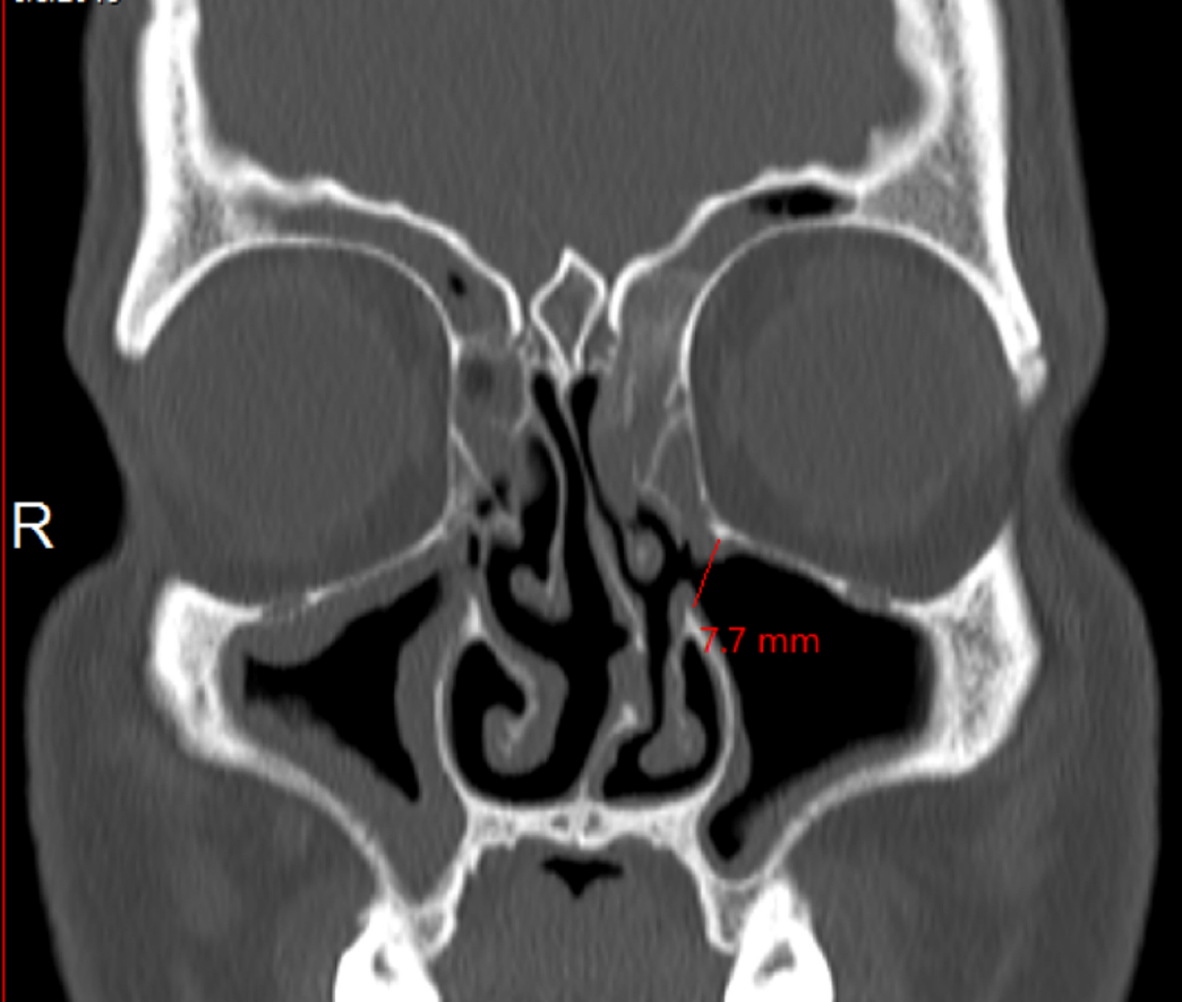
CT scan of paranasal sinus shows left AMO measuring 7.7 mm. CT: computed tomography, AMO: accessory maxillary ostium.

A 34-year-old male was presented to the ENT clinic complaining of chronic nasal obstruction and postnasal drip. Endoscopic examination of the nose showed: deviated nasal septum, bilateral hypertrophied inferior turbinate, and mucopurulent discharge from right middle meatus. Incidentally, a large AMO was found in the left anterior fontanelle (Figure 1). It was clear and patent. A computed tomography scan of the paranasal sinus was done that showed a sign of chronic rhinosinusitis. AMO was seen measuring 7 mm in the largest diameter (Figure 2). Eventually, the patient was booked for functional endoscopic sinus surgery (FESS) for chronic rhinosinusitis. 

## Discussion

This case showed a rare variation of AMO. It was uncommonly large compared to the reported AMO and located in a rare location (the anterior fontanelle). In the literature, the size of AMO varied from 0.5 mm to 5 mm [2]. Additionally, AMO can be present in unilateral, bilateral, single, or multiple in one side [2,6].

The presence of an AMO is reportedly more frequent in patients with chronic sinusitis [7]. Yenigun et al. reported that the presence of AMO was associated with an approximately threefold increase in the incidence of mucus retention cysts and a twofold increase in the incidence of mucosal thickening and maxillary sinusitis [8]. Yet, it is not clear whether the AMO is congenital or acquired, or whether resulted from maxillary sinusitis or leads to chronic rhinosinusitis. A possible mechanism for the development of accessory ostium is that in chronic rhinosinusitis, the maxillary sinus outflow obstructed with inflammation leads to accumulation of discharge which results in rupture of the fontanelle [9]. However, AMO reported in a normal individual making congenital entity another cause of AMO presence [1,4]. Drainage of the maxillary sinus depends on mucociliary clearance of the discharge that beat towards the natural ostium. When AMO present, the maxillary secretion can re-enter the sinus through the AMO lead to recirculation of the discharge [10]. Consequently, the patient may develop maxillary sinusitis or failure of chronic rhinosinusitis treatment. Thereby, it is important in the surgical treatment of maxillary sinusitis to open the natural ostium and connect it to the AMO to make one ostium to prevent recirculation of maxillary sinus mucus.

## Conclusions

Knowledge about different sinonasal anatomical variations is extremely essential to prevent surgical complications. AMO is one of the sinonasal anatomic variations. It is crucial to identify AMO and not confuse it with natural maxillary ostium. Additionally, It is important to address it during FESS and connect it to the natural maxillary ostium to prevent the recirculation of maxillary sinus mucus and reduce the risk of sinusitis recurrence.
